# The effects of restraint stress on ceramide metabolism disorders in the rat liver: the role of CerS6 in hepatocyte injury

**DOI:** 10.1186/s12944-024-02019-x

**Published:** 2024-03-02

**Authors:** Yichang Liu, Zhaoling Sun, Qiuli Sun, Li Wang, Chuan Wang, Yingmin Li, Chunling Ma, Weibo Shi, Guozhong Zhang, Yiming Dong, Xiaojing Zhang, Bin Cong

**Affiliations:** 1 Department of Forensic Medicine, Hebei Medical University, No. 361 Zhong Shan Rd, Shijiazhuang, 050017 Hebei China; 2 Department of Forensic Medicine, College of Medicine, Nantong University, Nantong, 226000 China; 3 Hebei Province Laboratory of Experimental Animal, Shijiazhuang, 050017 China; 4 Hainan Tropical Forensic Medicine Academician Workstation, Haikou, 571199 China

**Keywords:** Liver, Stress, Ceramide synthase 6, Ceramide, Mitochondria, Cytochrome c, AMPK, p38 MAPK

## Abstract

**Background:**

Stress is implicated in various pathological conditions leading to liver injury. Existing evidence suggests that excessive stress can induce mitochondrial damage in hepatocytes, yet the underlying mechanism remains unclear. Ceramide synthase 6 (CerS6)-derived C16:0 ceramide is recognised as a lipotoxic substance capable of causing mitochondrial damage. However, the role of CerS6 in stress has received insufficient attention. This study aimed to explore the involvement of CerS6 in stress-induced hepatic damage and its associated mechanisms.

**Methods:**

The rat restraint stress model and a corticosterone (CORT)-induced hepatocyte stress model were employed for in vivo and in vitro experimental analyses, respectively. Changes in mitochondrial damage and ceramide metabolism in hepatocytes induced by stress were evaluated. The impact of CORT on mitochondrial damage and ceramide metabolism in hepatocytes was assessed following CerS6 knockdown. Mitochondria were isolated using a commercial kit, and ceramides in liver tissue and hepatocytes were detected by LC–MS/MS.

**Results:**

In comparison to the control group, rats subjected to one week of restraint exhibited elevated serum CORT levels. The liver displayed significant signs of mitochondrial damage, accompanied by increased CerS6 and mitochondrial C16:0 ceramide, along with activation of the AMPK/p38 MAPK pathway. In vitro studies demonstrated that CORT treatment of hepatocytes resulted in mitochondrial damage, concomitant with elevated CerS6 and mitochondrial C16:0 ceramide. Furthermore, CORT induced sequential phosphorylation of AMPK and p38 MAPK proteins, and inhibition of the p38 MAPK pathway using SB203580 mitigated the CORT-induced elevation in CerS6 protein. Knocking down CerS6 in hepatocytes inhibited both the increase in C16:0 ceramide and the release of mitochondrial cytochrome c induced by CORT.

**Conclusions:**

CerS6-associated C16:0 ceramide plays a mediating role in stress-induced mitochondrial damage in hepatocytes. The molecular mechanism is linked to CORT-induced activation of the AMPK/p38 MAPK pathway, leading to upregulated CerS6.

**Supplementary Information:**

The online version contains supplementary material available at 10.1186/s12944-024-02019-x.

## Introduction

Threats can induce a non-specific psychobiological and neuroendocrine reaction, termed stress, which has been implicated in the pathogenesis of liver diseases in both clinical and experimental studies [1, 2]. Mitochondria, beyond their role in cellular energy production, play a crucial role in various cellular processes, including cell signaling, apoptosis, autophagy, antiviral immune responses, and the regulation of cell proliferation and development [3–5]. The severity of liver injury has been linked to mitochondrial respiratory dysfunction and oxidative stress [6]. Mitochondria are particularly vulnerable to the effects of the stress response [7]. Although stress-induced hepatic mitochondrial damage has been reported in recent years, the underlying mechanisms remain poorly understood [8, 9]. Under stress, activation of the hypothalamus–pituitary–adrenal cortex axis (HPA) leads to the release of excessive glucocorticoid (GC) hormones (cortisol in humans or corticosterone (CORT) in rodents) by the adrenal cortex [10]. Current research focuses on the mechanisms through which prolonged exposure to glucocorticoids affects neuronal mitochondrial dysfunction [11, 12], but insufficient attention has been given to the impact of glucocorticoids on liver mitochondrial function.

Ceramides, crucial bioactive sphingolipids, have been reported to influence mitochondrial respiratory chain function and participate in mitochondrial apoptosis, mitophagy, and mitochondrial dynamics [3, 4, 13–15]. Ceramides are produced through the N-acylation of sphingosine long-chain bases, a process regulated by ceramide synthase (CerS), also known as longevity assurance homolog (LASS) [16, 17]. CerS (CerS1-6) generates different acyl chain ceramides in mammals [16, 17]. Additionally, ceramides can be formed by the hydrolysis of sphingomyelin through sphingomyelinase [18]. Despite the critical role of CerS in regulating ceramide acyl chain length, little is known about its involvement in the stress response, leading to a limited understanding of the relationship between mitochondrial damage and disturbed ceramide metabolism during stress. C16:0 ceramide has been implicated in stress-induced injury in the liver and brain [19, 20]. CerS6-derived C16:0 ceramide is associated with hepatic mitochondrial β-oxidation and mitochondrial dynamics, contributing to insulin resistance, hyperglycemia, and obesity [15, 21, 22]. However, whether CerS6-derived C16:0 ceramide is linked to stress-induced mitochondrial damage in hepatocytes remains unknown.

Rodents undergo considerable psychological stress when subjected to restraint, resulting in behavioural changes such as anxiety and depression [23, 24]. Restraint stress (RS) is known to induce damage to the central nervous system, making it a widely adopted method in the exploration of the pathophysiological mechanisms and pharmacological treatment of depression [23–25]. The research group has undertaken a series of studies concentrating on the mechanisms underlying multiorgan damage triggered by restraint stress. Previous investigations have demonstrated that restraint stress induces pathological alterations in the myocardium,amygdala, hippocampus, and blood–brain barrier [26–29].

In the current study, the hypothesis posits that RS induces disturbances in rat hepatic ceramide metabolism, potentially leading to mitochondrial damage. This damage may be associated with the excessive accumulation of CORT, resulting in a specific CerS-mediated accumulation of mitochondrial ceramides. To elucidate this mechanism, the present study initially uncovered stress-induced hepatic mitochondrial injury and disruption of ceramide metabolism using a rat RS model. Subsequently, the CORT-induced hepatocyte stress model was employed to further explore the mechanism of CerS6-mediated C16:0 ceramide production in stress-induced mitochondrial injury in hepatocytes. This study presents the initial evidence that CerS6 and C16:0 ceramide contribute to stress-induced mitochondrial damage in hepatocytes.

## Materials and methods

### Animals and treatments

Male Sprague–Dawley rats (5–6 weeks old, weighing 160–180 g) were procured from Beijing Vital River Laboratory Animal Technology Co., Ltd. (Beijing, China) and maintained under standard laboratory conditions, as previously outlined [29]. The experimental procedures were sanctioned by the Local Committee of Animal Care, Use, and Protection of Hebei Medical University. Approval for all animal experiments was obtained from the Laboratory Animal Ethical and Welfare Committee of Hebei Medical University. As described in a preceding experiment, the rats were subjected to restraint using plastic tubes, commencing after one week of acclimatization [29]. In brief, the rats were randomly allocated into two groups: a control group and a RS group for 7 days. Rats in the RS group were restrained for 6 h daily and denied food and water during the restraint period. Simultaneously, when other rats were restrained, the control group was subjected to food and water deprivation. Prior to restraint each morning, rat weights were recorded. On the 7th day post-restraint, elevated plus maze (EPM) tests were promptly conducted following the restraint period. Subsequently, rats were anesthetized and sacrificed, and samples of blood and liver were collected.

### EPM test

The EPM test evaluated rat behavioral changes, with the instrument and experimental protocol detailed in a previous study [29]. Data regarding the percentage of time spent in open arms and entries into the open arms out of total entries were collected for statistical analysis.

### Enzyme-Linked Immunosorbent Assay (ELISA)

Serum CORT content was measured using a CORT ELISA kit (Arigo, ARG80652, Hsinchu, Taiwan, China) following the manufacturer's instructions. Enzymatic activity of alanine aminotransferase (ALT) and aspartate aminotransferase (AST) in serum was determined using ELISA kits (C009-2–1, C010-2–1, Nanjing Jiancheng Bioengineering Institute, Nanjing, China), and lactate dehydrogenase (LDH) was also measured (ab102526, Abcam, USA). All procedures strictly adhered to the manufacturer's instructions.

### Isolation of mitochondria from liver tissue

Mitochondria were isolated from fresh liver tissue using the Mitochondrial Isolation Kit (ab110168, Abcam, USA) according to the manufacturer's instructions. Liver tissue (50 mg) was homogenized in 2 ml of isolation buffer using a Dounce homogenizer. The isolated mitochondria were divided into two parts: one for Western blot analysis and the other for LC–MS/MS. Purity of the mitochondrial and cytoplasmic preparations was assessed by western blot using specific antibodies: anti-translocase of outer mitochondrial membrane 20 (TOMM20, mitochondrial marker), anti-voltage-dependent anion channel (VDAC, mitochondrial marker), and anti-calnexin (endoplasmic reticulum (ER) marker). Fifteen μg of cytoplasmic or mitochondrial lysate was loaded into the wells.

### Western blot analysis

**Fig. 1:**
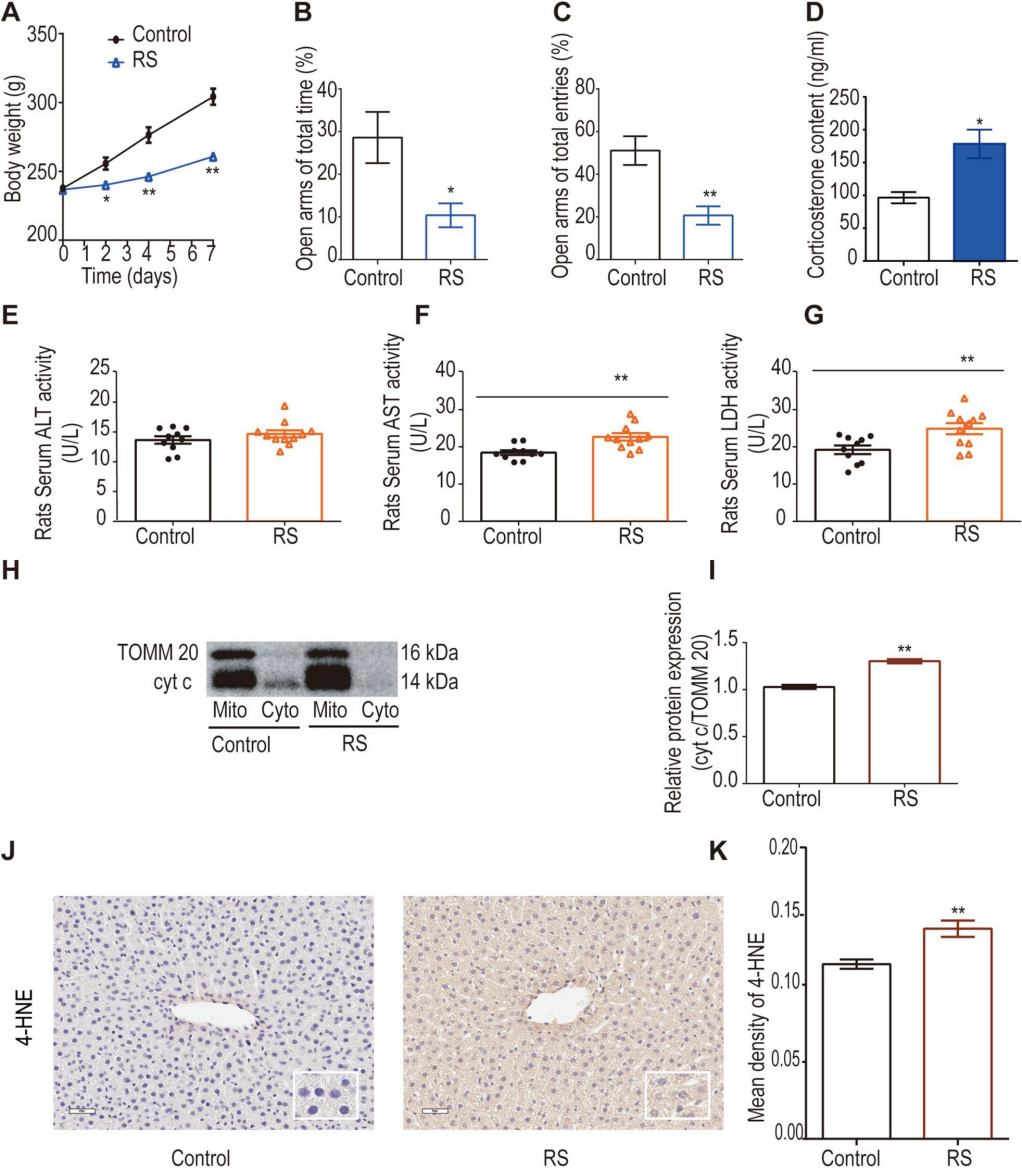
RS induces mitochondrial damage in the liver of rats. **A** Effects of RS on rat weight (*n* = 10–11). **B-C** Evaluation of anxiety-like behaviour in rats using EPM tests (*n* = 10–11). **D** Effects of RS on serum corticosterone levels in rats (*n* = 4–5). **E–G** Serum ALT, AST, and LDH levels were determined using commercial kits to assess changes in liver function due to RS (*n* = 10–11). **H-I** The cytoplasmic (Cyto) and mitochondrial (Mito) fractions were isolated utilising a Mitochondria Isolation Kit. Cytosolic and mitochondrial cyt c content was ascertained through western blot analysis. Mitochondrial cyt c was normalised to TOMM20 as an internal control (*n* = 5). **J-K** Immunohistochemical detection of 4-HNE in the liver and quantification with ImageJ software to evaluate the level of lipid peroxidation (*n* = 5). Data were analysed using Sidak's multiple comparisons test (body weight), Mann–Whitney test, or unpaired t-test. ^*^ *P* < 0.05, ^**^ *P* < 0.01, compared to the control group

The western blotting protocol followed procedures detailed in a prior experiment [29]. In brief, liver tissues and mitochondria were mechanically homogenized using RIPA lysis buffer (ab156034, Abcam, USA) containing a protease inhibitor cocktail (k1007, APExBIO Technology, Houston, USA), and a phosphatase inhibitor cocktail (k1012 + k1013, APExBIO Technology, Houston, USA). Following 15 min of incubation on ice, the lysates underwent centrifugation at 14,000 × g and 4 °C for 15 min, and the supernatants were collected. Protein concentrations were determined through the bicinchoninic acid (BCA) assay (TH269580, Thermo Fisher Scientific, Rockford, USA). Subsequent to 12% or 10% SDS polyacrylamide gel electrophoresis, proteins were transferred onto a PVDF membrane (Beyotime Biotechnology) by transmembrane and blocked with 5% nonfat milk or 5% BSA (for phosphorylated proteins) at 37 °C for 1 h. Membranes were incubated with the following antibodies: anti-cytochrome c (cyt c, 1:2000, ab133504), anti-TOMM20 (1:1000, ab186735), anti-α-Tubulin (1:1000, AF5012, Beyotime Biotechnology), anti-Ceramide synthase 2 (1:1000, ab227501), anti-LASS4 (1:500, AV36036, Sigma–Aldrich, St. Louis, USA), anti-LASS5 (1:1000, ab73289), anti-LASS6 (1:1000, ab115539), anti-VDAC (1:1000, D73D12, Cell Signaling Technology), anti-Calnexin (1:1000, ab133615), anti-GM130 (1:1000, ab52649), anti-p38 MAPK (1:500, 612,288, BD), anti-phospho-p38 MAPK (1:1000, 4511, Cell Signaling Technology), anti-AMPKα (1:1000, 5832, Cell Signaling Technology), and anti-Phospho-AMPKα (1:1000, 2535, Cell Signaling Technology), overnight at 4 °C, respectively. The antibodies mentioned were procured from Abcam (San Francisco, CA, USA), unless stated otherwise. After primary antibody incubation, membranes were further incubated with the IRDye 800 goat anti-rabbit antibody (1:10,000, forcyt c and TOMM20 only, Fig. 1H, Lincoln, NE, USA) or horseradish peroxidase (HRP)-conjugated secondary antibodies (1:1000, S1002, Hebei Report Bio&technology, Shijiazhuang, China) for 1 h at 37 °C. Detection employed ECL chemiluminescent substrate (Beyotime Biotechnology), and membranes were imaged with Azure C500 (Azure Biosystems, Dublin, CA, USA). The optical density of the blots was quantified using ImageJ 1.6 software (NIH, Bethesda, MD, USA).

### Immunohistochemistry

Liver sections designated for immunohistochemistry were fixed in 10% formalin, dehydrated through a graded ethanol series, and subsequently embedded in paraffin. Leica microtome was employed to obtain 5 μm thick paraffin sections. Prior to immunohistochemical staining, paraffin sections underwent deparaffinization with xylene. Immunohistochemistry procedures were conducted following previous methods [30]. In brief, microwave antigen retrieval was performed on paraffin sections, followed by permeabilization with 0.2% Triton X-100 in PBS for 25 min at room temperature. The tissues were then incubated with 3% hydrogen peroxide in PBS for 15 min, followed by a 1-h incubation with goat serum at room temperature. Subsequently, tissues were exposed to Anti-4-Hydroxynonenal (4-HNE) antibody (1:200, ab46545, Abcam) overnight at 4 °C. Following PBS rinses, sections were incubated with horseradish peroxidase (HRP)-conjugated biotin for 30 min (ZB-2301, Zhongshan Golden Bridge, Beijing, China). Afterward, tissues were treated with 3,3'-diaminobenzidine (DAB) for 3 min, stained with hematoxylin, and covered with coverslips. Negative controls involved substituting the primary antibodies with PBS. The slides were visualized using Leica Microsystems at a magnification of 400 × . The IHC Profiler plug-in, integrated with ImageJ software (Media CybernetiHOPE, Inc., Rockville, MD, USA), was utilized to analyse the immunohistochemical staining images following the provided operation instructions. The average optical density value was then obtained for semiquantitative analysis.

### Cell culture, induction, and treatment

The rat hepatocyte cell line BRL-3A (Identifier: CSTR:19,375.09.3101RATGNR10) was procured from the Cell Bank of the Chinese Academy of Sciences (Shanghai, China). Cells were cultured in RPMI 1640 medium (HyClone, Logan, UT, USA) supplemented with 10% FBS (Gibco, Grand Island, NY, USA) under standard conditions (37 °C, 5% CO_2_). Treatment was initiated when cells reached a confluence of 50–60%.

To assess mitochondrial damage in hepatocytes induced by stress, hepatocytes were treated with 50 μM CORT (NO. 16,063, Cayman Chemical, USA) diluted in 1640 medium for 24 h or 48 h after 12 h of serum deprivation. The vehicle group received treatment with 0.5% ethanol.

To investigate whether the p38 MAPK pathway is involved in the upregulation of CerS6 protein expression induced by CORT, hepatocytes were initially pretreated with 0.2% DMSO or 10 μM SB203580 (Adezmapimod, Cayman Chemical, USA) for 1 h after 12 h of serum deprivation. Subsequently, they were treated with 50 μM CORT or 0.5% ethanol for 24 h.

The scrambled control and CerS6 siRNA were synthesized by Shenggong Inc. (Shanghai, China). CerS6 siRNA sequences: sense (5'-3'): CCAAGCCAAUGGACCACAATT, antisense (5'-3'): UUGUGGUCCAUUGGCUUGGTT. Scrambled siRNA sequences: sense (5'-3'): UUC UCC GAA CGU GUC ACG UTT, antisense (5'-3'): ACG UGA CAC GUU CGG AGA ATT. Transfections of siRNA (40 nM) in hepatocytes were performed using Lipofectamine 8000 (C0533, Beyotime Biotechnology, Shanghai, China) following the manufacturer's instructions. Twenty-four hours after transfection, followed by 12 h of serum-free incubation, hepatocytes were treated with 50 μM CORT or 0.5% ethanol for 24 h. Subsequently, the cells were collected for western blot analysis or mitochondrial isolation. Transfection efficiency was assessed using western blotting.

### Isolation of mitochondria in hepatocytes

Mitochondria were isolated from hepatocytes using the Mitochondrial Isolation Kit (ab65320, Abcam) following the manufacturer's instructions. After treatment, cells were detached from the dishes through digestion with 0.25% trypsin (C0203, Beyotime Biotechnology, Shanghai, China). The hepatocytes (4 × 10^7^) were homogenized in 2 ml of Cytosol Extraction Buffer (with added protease inhibitor cocktail and DTT) using a Dounce homogenizer. The purity of the mitochondrial and cytoplasmic preparations was characterized similarly to the method employed in the animal experiments described previously. Cytoplasmic or mitochondrial lysate (15 μg) was added to the wells.

### Cellular immunochemistry staining

BRL-3A cells were cultured on coverslips. Following treatment, cells were fixed with 4% paraformaldehyde for 15 min. Subsequently, the cells were permeabilized with 0.2% Triton X-100 in PBS for 15 min at room temperature. The cell slides were then incubated with PBS containing 0.3% hydrogen peroxide for 3 min after being adequately rinsed in PBS. All subsequent procedures were carried out in accordance with the immunohistochemical methods mentioned above.

### Immunofluorescence

Immunofluorescence was performed as previously reported [31]. Mitochondria were stained by incubating cells with 250 nM Mito-Tracker™ Red (Molecular Probes) for 30 min. Following fixation with 4% paraformaldehyde for 15 min and permeabilization with 0.5% Triton X-100 in PBS for 10 min, the cells were incubated with Anti-4-HNE antibody (1:200) at 4 °C overnight after incubation with goat serum for 30 min at room temperature. Subsequently, the cells were incubated with the secondary antibody conjugated to goat anti-rabbit IgG (H + L) Highly Cross-Adsorbed Secondary Antibody Alexa Fluor 488 (PA5-16,891, Thermo Fisher Scientific, Rockford, USA). Cells were mounted with Fluoroshield Mounting Medium with DAPI (ab104129, Abcam). Images were captured using constant exposure settings with a Leica SP8 (Leica Microsystems GmbH, Wetzlar, Germany) confocal laser scanning microscope. For colocalization analysis, the Coloc 2 plug-in in Fiji software was used to generate Mander's coefficient for individual cells [32]. Six cells were randomly selected from each group for analysis, and the experiment was repeated three times.

### RNA isolation and RT–PCR

Total mRNA from liver tissue was isolated using the Animal Total RNA Isolation Kit (RE03011, Foregene, Chengdu, China) and quantified using a NanoDrop spectrophotometer. RNA integrity was assessed using 1% agarose gel electrophoresis. The mRNA was reverse transcribed into cDNA by the PrimeScript RT reagent Kit (RR047A, Takara, Japan) with gDNA Eraser following the manufacturer's instructions. The primer sequences are provided in Table 1. Amplification was performed using TB Green reagent, following the manufacturer's instructions, on a 7500 system. The fold change was calculated using the ΔΔ threshold cycle (ΔΔCT) method and normalized by geometric averaging of multiple internal control genes, as described previously [33, 34]. The relative mRNA expression of the target genes was normalized to the geometric mean of two housekeeping genes, tubulin beta 2a (TUBB2a) and ubiquitin C (Ubc), to facilitate comparisons between the groups. TUBB2a and Ubc were previously identified as stable housekeeping genes for assessing liver mRNA expression [35, 36].

**Table 1:** List of primers used in RT-PCR experiments

Gene	Forward primer sequence (5’-3’)	Reverse primer sequence (5’-3’)
TUBB2a	GCCTTCACCTCTTCTACAGCCAAC	TGCCATGCTCATCGCTTATCACC
Ubc	AGATCCAGGACAAGGAGGGCATC	TCTGAGGCGAAGGACCAGGTG
CerS2	GCCTTTGACTCCCTGACTTCA	GGGGTAGGGTGAGGGCATGTA
CerS4	GTGGCTGTGGCAGGAGACATA	GCAAGGCCACGAATCTCTCAA
CerS5	GGATGCTGTTCGAGCGATTTA	CATCCCAGTCCAGTTGCTTTGA
CerS6	CAGCGACACAGGAGTGGACAA	CGCACCATGAAGATGCAGAA

### LC–MS/MS

The lipid extraction method was conducted following previous protocols, with minor adjustments [3, 37–39]. Mitochondrial pellets were re-suspended in ice-cold PBS, and the supernatant was obtained by centrifugation after vortexing without visible precipitation. Mitochondrial protein concentrations were determined using the BCA assay (TH269580; Thermo Fisher Scientific, Rockford, USA). Subsequently, mitochondrial lipids were extracted using the following procedure. Initially, an internal standard solution (C17:0 ceramide) was added to the supernatant. The supernatant was then mixed with isopropanol/ethyl acetate (15:85, v/v), sonicated in ice water (800 Hz) for 5 min, and the upper organic phase was collected by centrifugation at 4000 × g and 4 °C for 10 min. The precipitate and the aqueous phase were combined with a solution of isopropanol, deionized water, and ethyl acetate (3:1:6, v/v/v). After sonication and centrifugation, the organic phase was collected again. This process was repeated, and the collected organic phases were mixed and placed on ice for 20 min. The upper organic layer was transferred to a glass tube, evaporated at room temperature under a continuous stream of nitrogen, and the dried residue was dissolved in methanol. Subsequently, 10 μL of the solution was injected into the column.

Ceramide standards (Avanti Polar Lipids, Alabaster, AL, USA; Table 2) were dissolved in methanol (sonicated if necessary) to a stock concentration of 0.1 mg/ml and stored at -20 °C. Solutions of each ceramide standard were prepared in methanol at 37 °C using sonication before LC–MS/MS detection.

**Table 2:** The precursor m/z values of ceramides

Ceramide	precursor m/z
C16:0 Ceramide (d18:1/16:0)	538.52
C18:0 Ceramide(d18:1/18:0)	566.55
C20:0 Ceramide(d18:1/20:0)	594.58
C22:0 Ceramide(d18:1/22:0)	622.62
C24:0 Ceramide(d18:2(4E,8Z)/24:0)	648.63
C24:1 Ceramide(d18:1/24:1(15Z))	648.62
C17:0 Ceramide(d18:1/17:0)	552.54

Quantification of ceramide species was performed using LC–MS/MS, as previously described [3, 37–39]. Analysis was conducted on a Q ExactiveTM combined quadrupole OrbitrapTM mass spectrometer (Thermo Fisher Scientific, Waltham, USA) with an Acquity UPLC column (BEH C18, 2.1 mm × 100 mm, Waters, MA, USA). Ceramides were separated using gradient elution with a flow rate of 0.3 mL/min. Solvent A was 0.1% methanoic acid–water (v/v), and solvent B was 0.1% methanoic acid-acetonitrile (v/v). The gradient program included solvent B at 20% (0–2 min), 20%-98% (2.01–15 min), 98% (15.01–42 min), and 98%-20% (42.01–45 min). Table 2 provides the precursor m/z values of all analyzed ceramides in positive ion mass spectrometry (ESI–MS) mode. Responses to the analyzed ceramides were optimized for a linear calibration range of 50–5000 ng/ml and a sample analysis time of 45 min.

### Statistical analysis

GraphPad Prism software package 6.0 (GraphPad Prism Software, Inc., California, USA) was utilised for the statistical analysis and graphical representation of data. Normal distribution of the data was assessed as a preliminary step. If the data exhibited a normal distribution, Student's t-test was applied. Alternatively, the Mann–Whitney test was employed. All values are presented as the mean ± standard error (SEM). Statistical significance was established at *P* ≤ 0.05.

## Results

### RS-induced mitochondrial damage in rat liver

Indicators of HPA axis activation in rodents encompass changes in weight, CORT levels, and behavioral responses [40, 41]. Findings reveal a significant reduction in body weight during RS (Fig. 1A, *P* < 0.05). In the EPM experiment, the decreased percentage of time spent in the open arms (Fig. 1B, *P* < 0.05) and the reduced number of open arm entries (Fig. 1C, *P* < 0.01) in the RS group suggest RS-induced anxiety-like behavior in rats. Moreover, RS leads to a substantial increase in serum CORT levels (Fig. 1D, *P* < 0.05). Collectively, these results indicate HPA axis activation and pronounced stress characteristics following restraint.

Subsequently, liver damage was assessed by measuring serum ALT, AST, and LDH levels using commercial kits. Although ALT levels (Fig. 1E) exhibited no significant changes, alterations in AST (Fig. 1F, *P* < 0.01) and LDH (Fig. 1G, *P* < 0.01) levels indicated RS-induced liver damage. The mitochondria, a pivotal target organelle in the stress response [7], were further examined for RS effects on liver mitochondria by evaluating the protein expression of mitochondrial and cytoplasmic cyt c. Results reveal a significant upregulation of mitochondrial cyt c (Fig. 1H-I, *P* < 0.01) in the liver of rats in the RS group compared to the control group, while changes in cytoplasmic cyt c lacked statistical significance (data not shown). To further elucidate the implications of RS-induced elevation of mitochondrial cyt c, alterations in hepatic 4-HNE were assessed. Mitochondrial cyt c can interact with the phospholipid tetralinoleoyl cardiolipin (L4CL) on the mitochondrial membrane, leading to the production of 4-HNE (a lipid peroxidation marker) [42]. Results demonstrate increased expression of hepatocyte 4-HNE in response to RS (Fig. 1J-K, *P* < 0.01). In conclusion, it is confirmed that RS induces mitochondrial damage in the rat liver, consistent with previous reports [8].

### CORT-induced mitochondrial damage in hepatocytes

**Fig. 2:**
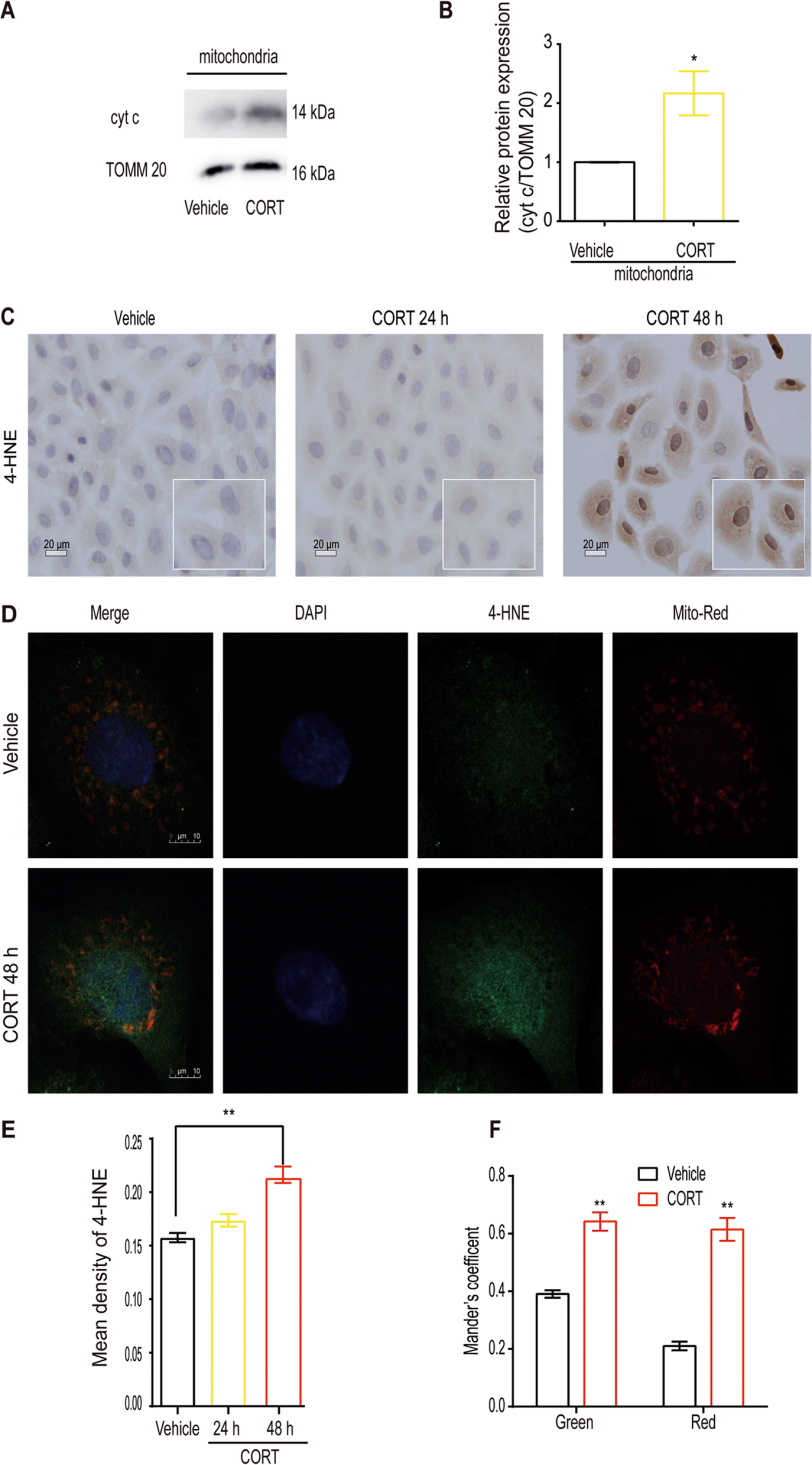
CORT induces mitochondrial damage in BRL-3A hepatocytes. Hepatocytes were treated with 50 μM CORT for 24 h or 48 h. **A-B** Mitochondrial cyt c in hepatocytes were determined by western blot (*n* = 3–4). **C, E** Immunohistochemical detection of 4-HNE and quantification with ImageJ software to assess lipid peroxidation levels. The region of interest (ROI) of the micrographs was enlarged for clear depiction (*n* = 3, scale bars: 20 μm). **D** Hepatocytes were stained with Mito-Tracker Red and 4-HNE, observed with a confocal fluorescence microscope (*n* = 3, scale bars: 10 μm). **F** Colocalization analysis was determined by calculating Mander’s coefficient using Coloc 2 in Fiji software (*n* = 3, 6 cells per experiment). Data were analysed by the Mann–Whitney test or unpaired t-test. ^*^ *P* < 0.05, ^**^ *P* < 0.01, compared to the vehicle group

To replicate the effect of CORT accumulation during stress on hepatocytes, hepatocytes were treated with 50 μM CORT. Results indicate that, compared with the vehicle group, CORT induces a significant increase in mitochondrial cyt c expression (Fig. 2A, B, *P* < 0.05) and 4-HNE expression (Fig. 2C, E, *P* < 0.01) in hepatocytes. Furthermore, the increase in mitochondrial cyt c and 4-HNE expression occurs chronologically. To further elucidate the mitochondrial damage caused by CORT, the mitochondria were labeled with Mito-Tracker, and the expression of 4-HNE within the mitochondria was evaluated using Mander's coefficient. The colocalization results indicate that the increase in 4-HNE induced by CORT is primarily localized in the mitochondria of hepatocytes (Fig. 2D, F, *P* < 0.01) but is not exclusively limited to mitochondria (Fig. 2D). Therefore, these data provide evidence of mitochondrial damage in hepatocytes induced by CORT.

### RS induces upregulation of hepatic CerS5 and CerS6 expression, resulting in mitochondrial C16:0 ceramide accumulation

**Fig. 3:**
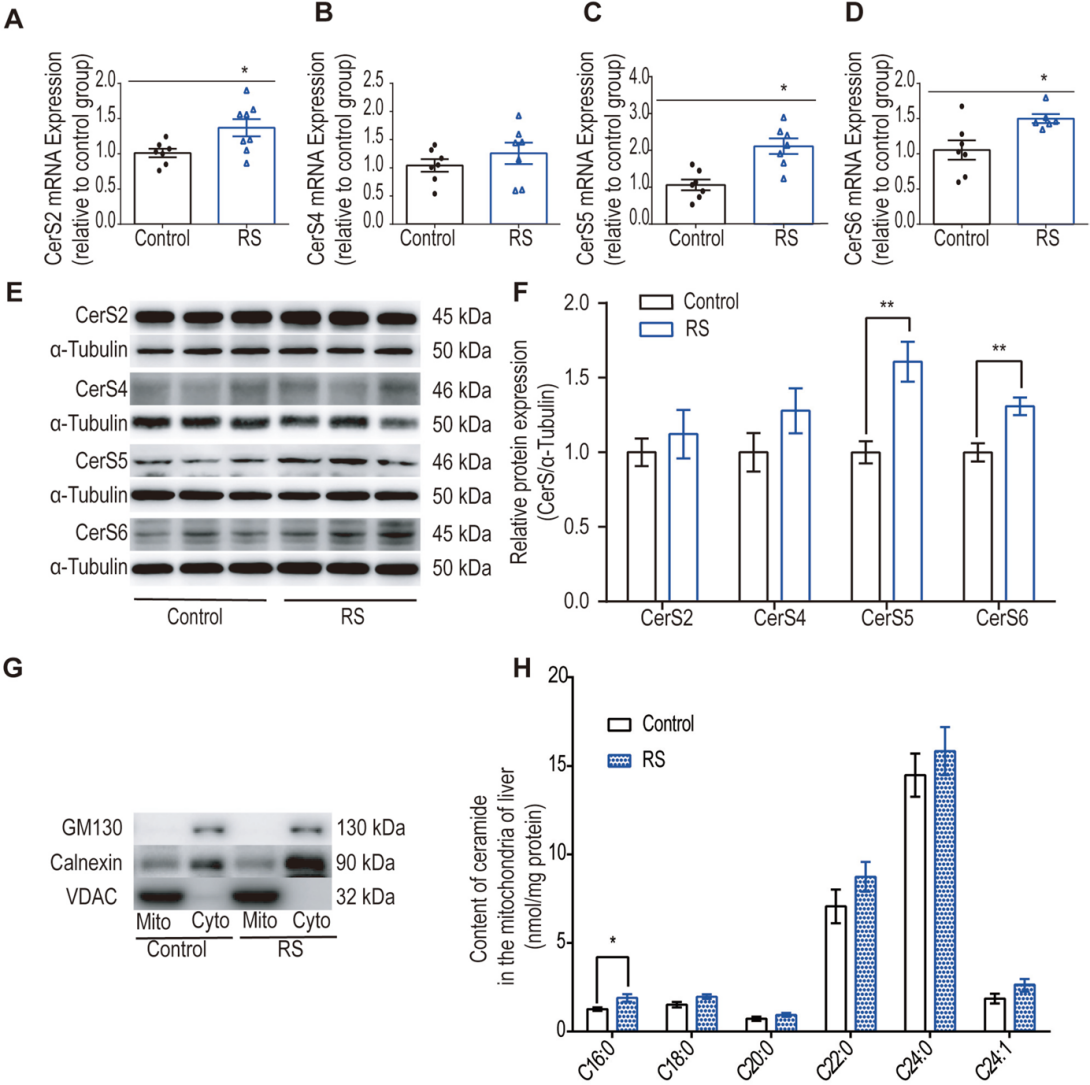
Effects of RS on rat liver CerSs and mitochondrial ceramides. **A-D** RT-PCR results demonstrated that RS induced an increase in CerS2, CerS5, and CerS6 in the liver (*n* = 6–8). Values were normalised to the geometric mean of two housekeeping genes (TUBB2a and Ubc) and presented as fold change relative to control values, as evaluated by the ΔΔCT method. **E–F** Western blot results revealed that RS increased the protein expression of CerS5 and CerS6 in the liver (*n* = 3–4). **G** Western blotting was employed to detect the contents of VDAC (a mitochondrial outer membrane marker), calnexin (an ER marker), and GM130 (a Golgi marker) in both isolated mitochondria and cytoplasm to assess the purity and integrity of the isolated mitochondria. **H** RS induced an increase in C16:0 ceramide content in isolated mitochondria (*n* = 5). Ceramide species content was measured by LC–MS/MS. Data were analysed by the Mann–Whitney test or unpaired t-test. ^*^ *P* < 0.05, ^**^ *P* < 0.01, compared to the control group

In the liver, CerS exist in four types: CerS2 generating C22-C24 ceramides, CerS4 producing C18-C20 ceramides, and CerS5 and CerS6 generating C14-C16 ceramides [16]. Findings reveal that RS led to an increase in both the mRNA (Fig. 3C-D, *P* < 0.05) and protein (Fig. 3E-F, *P* < 0.01) levels of CerS5 and CerS6, accompanied by elevated mRNA expression of CerS2 (Fig. 3A, *P* < 0.05). Using LC–MS/MS, ceramide content in isolated liver mitochondria was assessed. The results indicated that, while C24:0 ceramide was the most abundant species in crude mitochondria of liver tissue, RS significantly increased C16:0 ceramide content (Fig. 3H, *P* < 0.05).

To assess the purity of the isolated mitochondria, VDAC, a protein located on the outer membrane of mitochondria [43], was examined via western blotting. VDAC was predominantly expressed in isolated mitochondria, minimally in the cytoplasm (Fig. 3G). The endoplasmic reticulum (ER) is the primary organelle responsible for ceramide production, while the Golgi apparatus is the crucial compartment where ceramide undergoes modification by chemical groups [44–47]. Analysis of Golgi marker (GM130) indicated the absence of Golgi contamination in isolated mitochondria. However, the presence of calnexin, an ER marker, in both the cytosolic fraction and isolated mitochondria suggested the presence of crude mitochondria containing mitochondrial-associated membranes (MAMs) (Fig. 3G).

In summary, these results suggest a link between RS-induced upregulation of CerS5 and CerS6 and the metabolic accumulation of C16:0 ceramide in mitochondria.

###  CORT regulates CerS6 via the AMPK/p38 MAPK pathway in the liver during RS

**Fig. 4:**
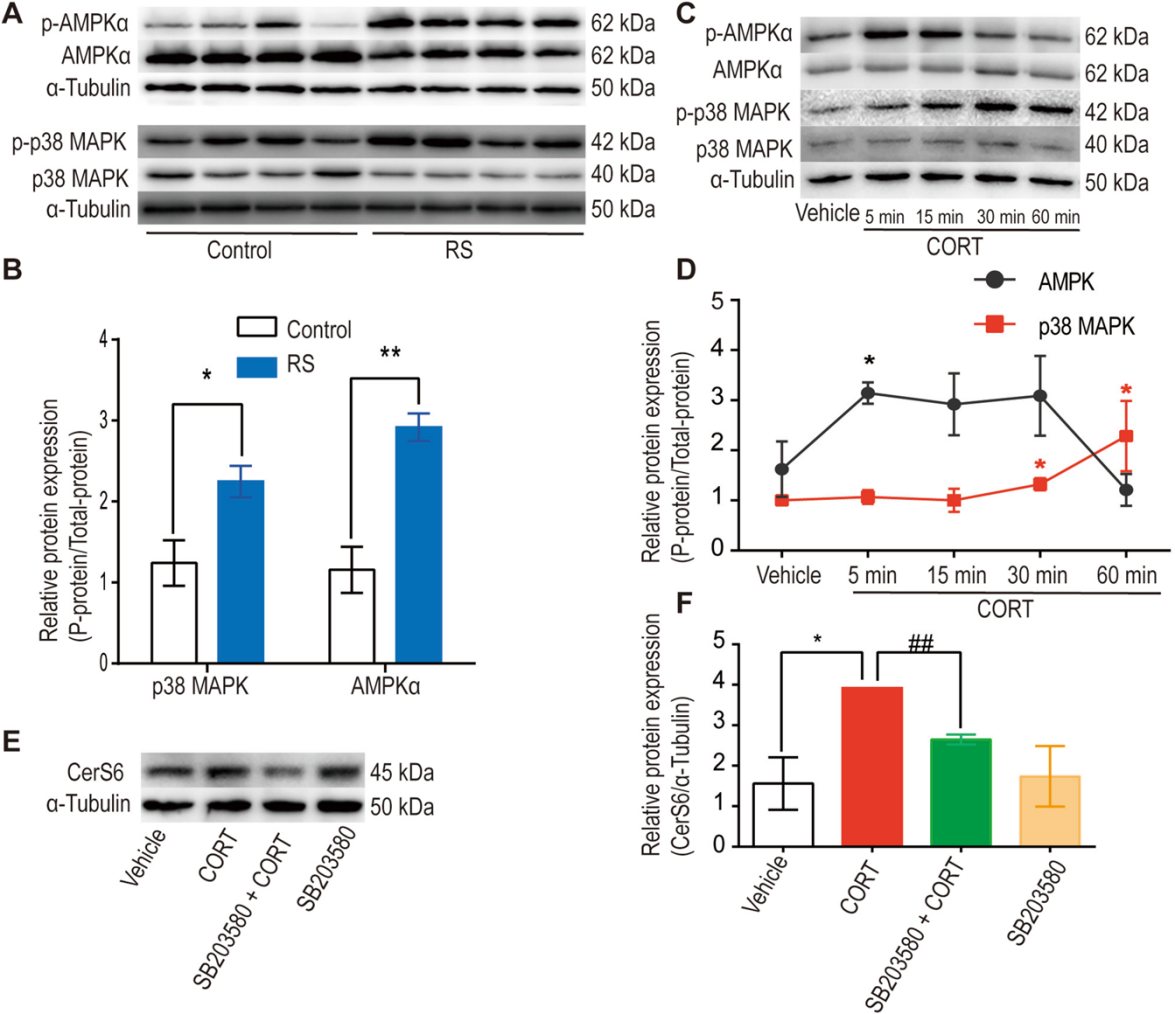
The AMPK/p38 MAPK pathway mediates the upregulation of CerS6 during stress. **A-B** RS induces phosphorylation of AMPK and p38 MAPK in the livers of rats (*n* = 4). Data were analysed by the unpaired t-test. ^*^ *P* < 0.05, ^**^ *P* < 0.01, compared to the control group. **C-D** CORT activates the AMPK/p38 MAPK pathway in hepatocytes in a time-dependent manner. Hepatocytes were treated with CORT (50 μM) or 0.5% alcohol (vehicle), ^*^ *P* < 0.05, compared to the vehicle group. **E–F** Inhibition of the p38 MAPK pathway by SB203580 reversed the upregulation of CerS6 protein expression induced by CORT. Hepatocytes were incubated with SB203580 (10 μM) for 0.5 h and then incubated with serum-free medium containing CORT (50 μM) or 0.5% alcohol (vehicle) for 24 h. Data were analysed by the Mann–Whitney test or unpaired t-test. *n* = 3–4, ^*^ *P* < 0.05, compared to the vehicle group. ^##^ *P* < 0.01, compared to the CORT group

To unravel the signaling pathways of CerS6 during stress, classical signaling pathways involved in lipid metabolism and stress, including the AMPK/p38 MAPK pathway, were investigated. RS induced AMPK and p38 MAPK phosphorylation in the liver (Fig. 4A, B, *P* < 0.05). In vitro, CORT treatment swiftly induced AMPK/p38 MAPK activation, with AMPK activation preceding p38 MAPK activation (Fig. 4C, D, *P* < 0.05). AMPK was identified as the upstream molecule responsible for CORT-induced p38 MAPK activation, aligning with prior reports [48]. Using SB203580 to inhibit p38 MAPK catalytic activity, the interplay between p38 MAPK and CerS6 in stress was explored. CORT upregulated CerS6 protein expression (Fig. 4E, F, *P* < 0.05, Vehicle *vs.* CORT), and SB203580 pretreatment inhibited the upregulation of CerS6 by CORT (Fig. 4E, F, *P* < 0.05, CORT vs. SB203580 + CORT). These findings suggest that CORT regulates CerS6 through the AMPK/p38 MAPK pathway, indicating a correlation with RS-induced CerS6 upregulation.

### CerS6 mediates CORT-induced mitochondrial C16:0 ceramide production and subsequent hepatocyte mitochondrial damage

**Fig. 5:**
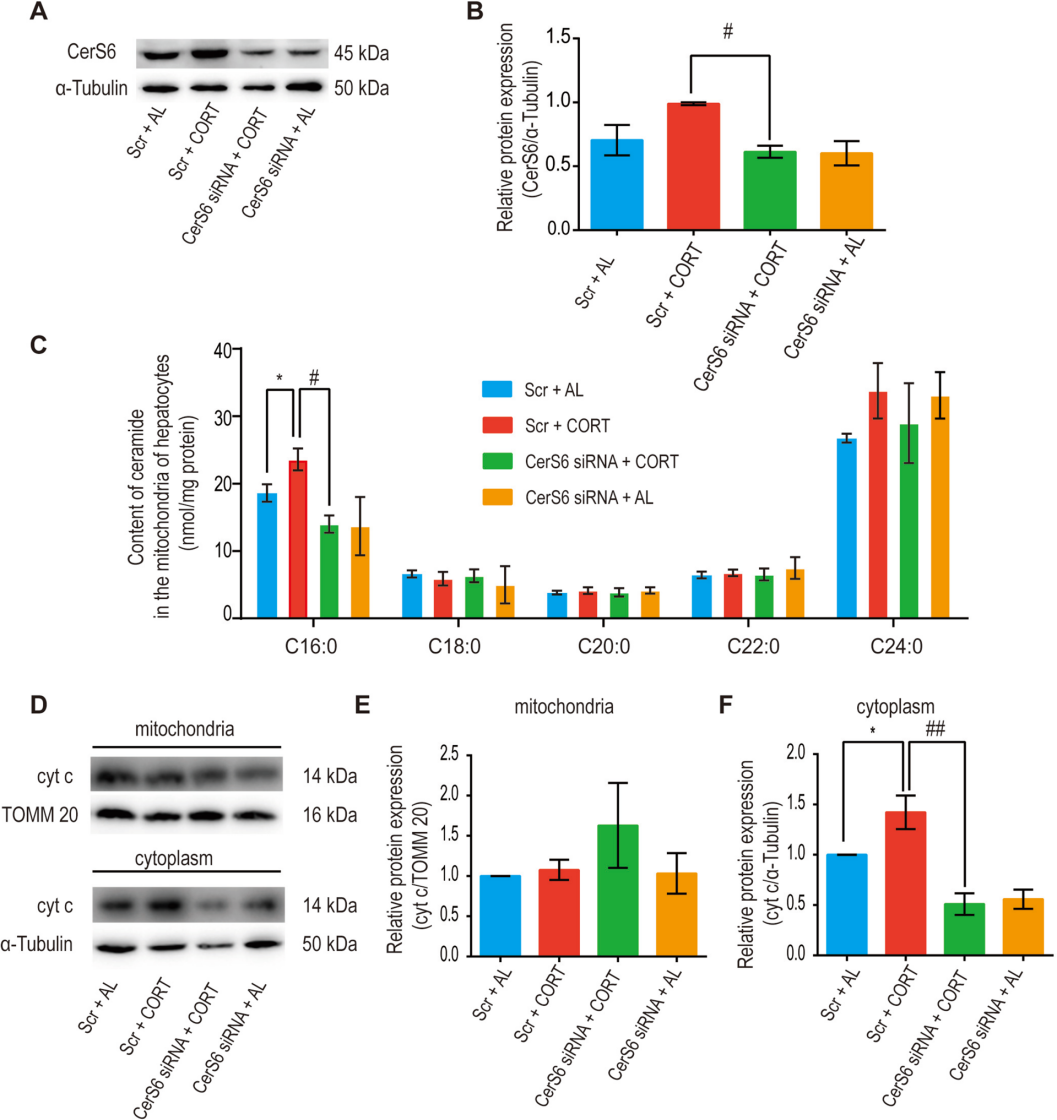
CORT induces the accumulation of mitochondrial C16:0 ceramide and mitochondrial damage through a CerS6-dependent pathway. Hepatocytes were transfected with scramble control (Scr) or CerS6 siRNA for 24 h. They were then cultured in serum-free medium for 12 h and treated with CORT (50 μM) or 0.5% alcohol (AL) for 24 h. Alcohol was used as a solvent control. **A-B** Treatment with CerS6 siRNA effectively inhibited the protein expression of CerS6. **C** The increase in mitochondrial C16:0 ceramide induced by CORT was reversed by CerS6 knockdown. **D-F** The release of cyt c from mitochondria into the cytoplasm induced by CORT was repressed by CerS6 knockdown. Data were analysed by the Mann–Whitney test or unpaired t-test. *n* = 3–4, ^*^ *P* < 0.05, compared to the Scr + AL group. ^#^ *P* < 0.05, ^##^ *P* < 0.01, compared to the Scr + CORT group

While both CerS5 and CerS6 have the capacity to generate C16:0 ceramide, recent research indicates that CerS6, rather than CerS5, predominantly regulates C16:0 ceramide in liver mitochondria and MAMs [15]. Investigations support the association between CerS6 upregulation and the concurrent increase in C16:0 ceramide induced by RS. To delve into CerS6's role in stress, we employed siRNA to knock down CerS6 in vitro, subsequently assessing various ceramide species in crude hepatocyte mitochondria through LC–MS/MS. Results revealed that CORT induced the upregulation of CerS6 protein expression (Fig. 5A, B, *P* = 0.06, Scr + AL *vs.* Scr + CORT), accompanied by an elevation in mitochondrial C16:0 ceramide (Fig. 5C, *P* < 0.05, Scr + AL *vs.* Scr + CORT). The CORT-induced upregulation of CerS6 protein expression and increased C16:0 ceramide were mitigated following CerS6 siRNA treatment (Fig. 5A-C, *P* < 0.05, Scr + CORT *vs.* CerS6 siRNA + CORT). These findings suggest that CORT influences hepatocyte mitochondrial C16:0 ceramide production through the regulation of CerS6.

In response to apoptotic stimuli, increased mitochondrial C16:0 ceramide formation has been linked to ceramide channel formation, facilitating the release of cyt c and other proapoptotic proteins from mitochondria into the cytosol, thereby initiating apoptosis [49]. To clarify whether mitochondrial C16:0 ceramide production triggers mitochondrial damage, cyt c levels were examined in both mitochondria and cytoplasm. Results indicated that CORT induced a significant increase in cytoplasmic cyt c (Fig. 5D, F, *P* < 0.05, Scr + AL *vs.* Scr + CORT), and this effect was attenuated after CerS6 knockdown (Fig. 5D, F, *P* < 0.01, Scr + CORT *vs.* CerS6 siRNA + CORT). The purity of mitochondrial isolates was assessed through western blotting of mitochondrial markers TOMM20 and VDAC in isolated mitochondrial and cytoplasmic fractions. Notably, mitochondrial isolation using a widely used kit theoretically ensures cytoplasmic components devoid of mitochondria; however, over-homogenization during the process may have compromised mitochondrial membrane integrity, leading to the release of mitochondrial components. This could explain the low expression of VDAC and TOMM20 in some cytoplasmic lysates by western blotting (Supplementary Fig. S1), potentially influencing result interpretation. In conclusion, findings suggest that both CORT-induced mitochondrial C16:0 ceramide accumulation and subsequent mitochondrial damage in hepatocytes are CerS6-dependent.

## Discussion

In recent years, CerS-derived ceramide has gained recognition as a contributor to mitochondrial dysfunction in hepatocytes in diabetes and obesity [15, 50]. However, the role of CerS-mediated ceramide production during stress has not been previously explored. This study establishes that CerS6-associated C16:0 ceramide acts as a mediator of mitochondrial damage in hepatocytes exposed to CORT during RS, and the AMPK/p38 MAPK pathway governs the regulation of CerS6 by CORT.

Clinical research has linked increased psychological stress to the pathological conditions of various liver diseases [51]. Moreover, animal experiments have demonstrated stress-induced liver mitochondrial dysfunction [2, 52]. Consistent with prior findings, the study indicates that stress induces mitochondrial damage in the liver, integrating results from in vivo and in vitro experiments. Intriguingly, RS led to an in vivo increase in mitochondrial cyt c, while the in vitro results were not entirely consistent. Instead, it was observed that CORT induced a rise in cytoplasmic cyt c, implying the release of mitochondrial cyt c. This paradoxical outcome can be explained by the dynamic alterations of cyt c during stress. Under apoptotic stimuli, mitochondrial cyt c initially increases and is subsequently released into the cytoplasm, activating downstream signaling and initiating apoptosis [53–55]. Additionally, studies have reported that cyt c directly acts on cell membranes or mitochondrial membranes, generating 4-HNE (a lipid peroxidation marker) and causing mitochondrial membrane damage [42]. Results indicate that the alteration in 4-HNE aligns with the dynamic changes in cyt c both in vivo and in vitro. In summary, these findings underscore the pivotal role of cyt c as a mediator of RS-induced mitochondrial damage. While previous studies have reported that mitochondrial cyt c release during apoptosis necessitates ceramide channels formed in the outer mitochondrial membrane [56], it remains unexplored whether ceramide is involved in mitochondrial cyt c release during stress.

Ceramide, a family of sphingolipids, comprises a sphingoid base linked to a fatty acid through an amide bond, featuring varying chain lengths. Various ceramide species have been identified in mammals [57, 58]. Recent findings indicate that stress triggers the production of C16:0 ceramide in mouse liver [19]. However, the precise role of ceramides in stress-induced liver injury remains unclear. Given the potential involvement of ceramides in stress-induced mitochondrial damage, alterations in the content of distinct ceramide species in rat liver mitochondria in response to RS were investigated. The results revealed a significant increase in C16:0 ceramide content in mitochondria induced by RS.

The distribution of CerSs is tissue and cell-specific, with each tissue or cell exhibiting a unique distribution of sphingolipid acyl chain lengths [16, 17]. To further ascertain that CORT primarily affects hepatocytes, leading to mitochondrial C16:0 ceramide production during stress, cellular experiments were conducted. It is noteworthy that stress-induced mitochondrial dysfunction is believed to be associated with the excessive release of glucocorticoids [59, 60]. The research provides direct evidence that C16:0 ceramide mediates CORT-induced mitochondrial damage.

Recent investigations have highlighted the pivotal role of mitochondrial ceramide accumulation in the early stages of apoptosis, involving enzymatic reactions facilitated by enzymes like acid sphingomyelinase (ASM) and CerS [61]. Indeed, Gulbins et al. have validated the activation of the ASM/ceramide pathway by stress in the brain and liver [19, 20]. The study extends this understanding by providing evidence that stress also impacts CerSs. CerS comprises a group of isoenzymes crucial for determining ceramide acyl chain lengths [16]. Despite both CerS5 and CerS6 capable of generating C16:0 ceramide, only CerS6 regulates C16:0 ceramide in mitochondria and MAMs, not CerS5 [15]. Additionally, CerS6 is expressed in both the endoplasmic reticulum and mitochondria of hepatocytes [15]. Thus, the hypothesis is that the accumulation of mitochondrial C16:0 ceramide during RS might be regulated by CerS6. Findings suggest that C16:0 ceramide, produced by CerS6, mediates stress-induced mitochondrial damage. Knockdown of CerS6 reduced CORT-induced mitochondrial C16:0 ceramide accumulation and reversed the release of mitochondrial cyt c induced by CORT.

Previous evidence indicates that CerS6-dependent mitochondrial C16:0 ceramide is involved in regulating mitochondrial dynamics and insulin resistance in obese rats [15, 22]. Another study showed that CerS6 knockdown with siRNA suppressed mitochondrial apoptosis induced by folate deficiency in vitro [62]. Hence, it is speculated that C16:0 ceramide, produced by CerS6, is a stable component independently affecting the mitochondrial function of hepatocytes, irrespective of the inducing factors. Further research and diverse models are required to validate this perspective. Notably, the mechanism through which CORT induces CerS6 to produce C16:0 ceramide, leading to hepatocyte injury, likely involves ER stress. The ER, a critical organelle for ceramide metabolism, is closely connected to mitochondria through its outer membranes (MAMs) [47]. The CORT-induced increase in mitochondrial C16:0 ceramide in hepatocytes in this study cannot exclude MAM-derived C16:0 ceramide. Previous studies have demonstrated that ceramide is a significant factor leading to ER stress, and CerS6 knockdown with siRNA prevents palmitate-induced ER stress in intestinal epithelial cells [63]. In a recent study, McNally et al. [64] showed that lipotoxicity-induced ER stress signaling through CerS2 stimulates long-chain ceramide biosynthesis and secretion in skeletal muscle. These findings suggest that the interaction between ceramide and ER stress signaling is a crucial molecular mechanism in lipotoxic injury, warranting further exploration in stress models.

To date, research has predominantly delved into the molecular mechanisms involving CerS6 itself, with limited exploration of its upstream signaling pathways. In the study, the activation of the AMPK/p38 MAPK pathway by RS was observed in vivo. Subsequently, the regulatory impact of CORT on the AMPK/p38 MAPK pathway was corroborated in vitro. Notably, the elevated expression of CerS6 induced by CORT was reversed by SB203580, a well-established inhibitor of the p38 MAPK pathway. These findings suggest the involvement of the AMPK/p38 MAPK pathway in the regulation of CerS6 during stress.

Existing evidence indicates that CerS6 is directly regulated by p53 in apoptosis [62, 65]. Moreover, the AMPK/p38 MAPK pathway serves as an upstream signaling pathway for p53 [66–68]. The question of whether the AMPK/p38 MAPK pathway regulates CerS6 during stress through a direct impact on p53 remains a subject for further investigation.

### Study strengths and limitations

Previous research within the group has substantiated that RS induces detrimental effects on the heart and blood–brain barrier [26, 29]. Furthermore, C16:0 ceramide has been identified as a lipotoxic mediator responsible for heart and brain damage [69–71]. Notably, hepatocytes are recognized as significant contributors to circulating ceramide levels [72]. This experiment has provided clarity, establishing C16:0 ceramide as the instigator of stress-induced hepatocyte damage. This foundational understanding paves the way for exploring the intricate communication mechanisms between the liver, heart, and brain during stress.

However, a noteworthy limitation of this study is the existing evidence suggesting that CerS6 may have sex-dependent effects on liver metabolism [73]. Given that all the rats used in this experiment were male, further investigations are warranted to determine the applicability of these findings to female rats.

## Conclusions

The current study underscores the pivotal role played by CerS6 in the accumulation of mitochondrial C16:0 ceramide in hepatocytes during restraint stress in rats. Moreover, it provides preliminary evidence supporting the idea that mitigating stress-induced hepatocellular injury in rats can be achieved by inhibiting the CORT-induced upregulation of CerS6. The impact of stress on human health is increasingly gaining attention in medical practice, and the effects of stress on the liver have been associated with a variety of diseases, including hepatitis, non-alcoholic fatty liver disease, and liver tumors. As a noninvasive stressor, restraint stress effectively simulates the psychological stress associated with "uncontrollable" crowding and compulsion in human life. Rodent models of restraint stress are widely employed in preclinical studies for drug development. Both CerS6 and C16:0 ceramide emerge as potential targets for the prevention and treatment of stress-induced hepatocellular injury. Treatment of hepatocytes with CerS6 siRNA proves effective in reducing C16:0 ceramide levels in mitochondria and attenuating hepatocyte injury, providing a solid foundation for the development of siRNA drugs to address liver injury. Additionally, the exploration of commercially available drugs specifically targeting CerS6 represents a promising avenue for research and development, with these drugs being candidates for testing in preclinical models. In conclusion, the study suggests that CerS6 holds promise as a therapeutic target for hepatoprotection under stress and has considerable potential for clinical application.

### Supplementary Information

**Additional file 1: Figure S1.** Purity assessment of isolated mitochondria from hepatocytes. Western blotting was employed to quantify the levels of two mitochondrial markers, VDAC and TOMM20, in both the isolated mitochondria and the cytoplasm. The proteins detected on a single PVDF membrane also included cyt c and α-Tubulin.

## Data Availability

The datasets used and/or analyzed during the current study are available from the corresponding author upon reasonable request.

## Abbreviations

CerS: Ceramide synthase
CORT: Corticosterone
AMPK: AMP-activated protein kinase
p38 MAPK: P38 mitogen-activated protein kinase
HPA: Hypothalamus-pituitary-adrenal
GC: Glucocorticoid
LASS: Longevity assurance homolog
RS: Restraint stress
EPM: Elevated plus maze
ELISA: Enzyme-linked immunosorbent assay
ALT: Alanine aminotransferase
AST: Aspartate aminotransferase
LDH: Lactate dehydrogenase
Cyt c: Cytochrome c
VDAC: Voltage-dependent anion channel
TOMM20: Translocase of outer mitochondrial membrane 20
4-HNE: 4-Hydroxynonenal
TUBB2a: Tubulin beta 2a
Ubc: Ubiquitin C
MAM: Mitochondria-associated membrane
ER: Endoplasmic reticulum
ASM: Acid sphingomyelinase
