# Supplementary figures and images for: The effects of restraint stress on ceramide metabolism disorders in the rat liver: the role of CerS6 in hepatocyte injury
Source: Lipids Health Dis. 2024 Mar 2;23:68. doi: 10.1186/s12944-024-02019-x (PMC10908211; doi:10.1186/s12944-024-02019-x)

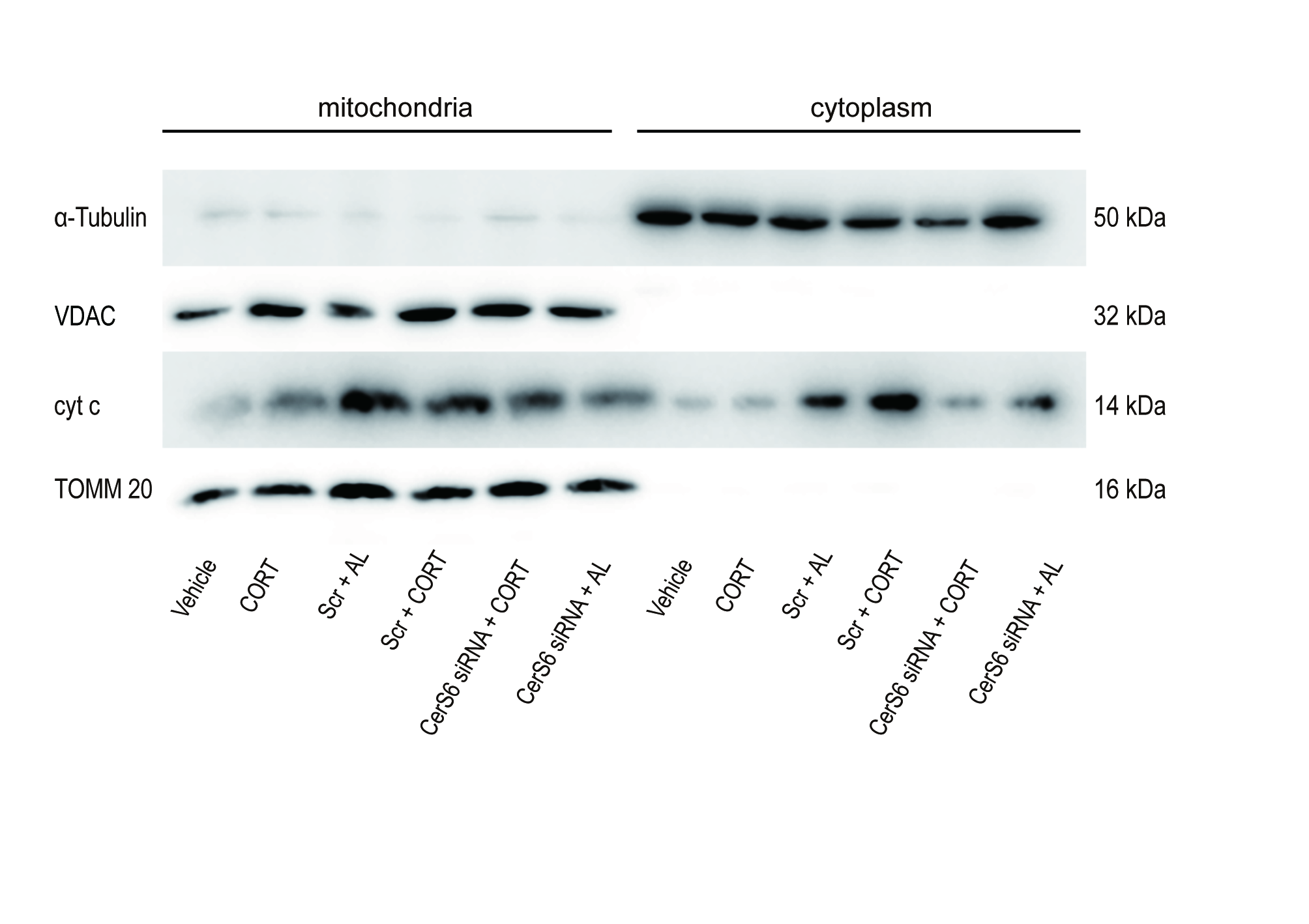

Supplement: Supplementary file 1 — Additional file 1: Figure S1. Purity assessment of isolated mitochondria from hepatocytes. Western blotting was employed to quantify the levels of two mitochondrial markers, VDAC and TOMM20, in both the isolated mitochondria and the cytoplasm. The proteins detected on a single PVDF membrane also included cyt c and α-Tubulin. [file 12944_2024_2019_MOESM1_ESM.tif]
